# Can TEMPO-Oxidized Cellulose Nanofibers Be Used as Additives in Bio-Based Building Materials? A Preliminary Study on Earth Plasters

**DOI:** 10.3390/ma16010074

**Published:** 2022-12-21

**Authors:** Paola Gallo Stampino, Laura Riva, Marco Caruso, Imran Abdul Rahman, Graziano Elegir, Daniele Bussini, Javier Marti-Rujas, Giovanni Dotelli, Carlo Punta

**Affiliations:** 1 Department of Chemistry, Materials, and Chemical Engineering “G. Natta” and INSTM Local Unit, Politecnico di Milano, 20133 Milan, Italy; 2 Materials Testing Laboratory, Politecnico di Milano, 20133 Milan, Italy; 3 Innovhub Stazioni Sperimentali per l’Industria S.r.l., Via Giuseppe Colombo 83, 20133 Milan, Italy

**Keywords:** cellulose nanofibers, raw earth, cellulose-based additives, sustainability, earth plaster

## Abstract

Interest towards cellulose nanofibers obtained from virgin and waste sources has seen a significant growth, mainly thanks to the increasing sensitivity towards the concept of circular economy and the high levels of paper recycling achieved in recent years. Inspired by the guidelines of the green building industry, this study proposes the production and characterization of TEMPO-oxidized and homogenized cellulose nanofibers (TOHO CNF) from different sources and their use as additives for earth plasters on two different raw earth samples, characterized by geotechnical laboratory tests and mineralogical analysis: a high-plasticity clay (T2) and a medium-compressibility silt (ABS). Original sources, including those derived from waste (recycled cardboard and paper mill sludge), were characterized by determining chemical content (cellulose versus ashes and lignin) and fiber morphology. TOHO CNF derived from the different sources were compared in terms of nanofibers medium diameter, crystallinity degree, thermal decomposition and oxidation degree, that is the content of carboxylic groups per gram of sample. Then, a preliminary analysis of the influence of CNF on earth plasters is examined. Adhesion and capillary absorption tests highlighted the effect of such nanofibers on blends in function of two factors, namely the cellulose original source and the oxidation degree of the fibers. In particular, for both earth samples, T2 and ABS, a significant increase in adhesion strength was observed in the presence of some TOHO CNF additives. As far as capillary sorption tests, while an undesired increase in water adsorption was detected for T2 compared to the control, in the case of ABS, a significant reduction in water content was measured by adding TOHO CNF derived from recycled sources. These results pave the way for further in-depth investigation on the role of TOHO CNF as additives for earth plasters.

## 1. Introduction

Traditional buildings and the whole construction sector together account for a large amount of the total greenhouse gas (GHG) emissions and energy consumption—38% and 35%, respectively [1]. Although there has been a decreasing trend in GHG emissions since 2005 in Europe, the EU 2030 emissions target still requires a strong effort in the way of renovation of the building stock and a drastic change in the paradigms of energy use as well as of construction materials [2]. The Intergovernmental Panel on Climate Change confirms that mitigation strategies for cities could include “bio-based building materials, permeable surfaces, green roofs, trees, green spaces, rivers, ponds and lakes” [3]. The development of more efficient building designs and more sustainable alternatives must be fostered to reduce the environmental burdens associated, as clearly indicated by the recently amended European directives on energy performance of buildings (Directive (EU) 2018/844 of the European Parliament and of the Council of 30 May 2018 amending Directive 2010/31/EU on the energy performance of buildings and Directive 2012/27/EU on energy efficiency) and on energy efficiency (Directive (EU) 2018/2002 of the European Parliament and of the Council of 11 December 2018 amending Directive 2012/27/EU on energy efficiency). Raw earth-based materials are gaining increasing attention thanks to the numerous advantages in terms of sustainability [4,5,6] including the reduced cost and energy needed for the production, transportation, and handling, as the worldwide availability of large quantities of clayish earth is associated with an abridgment of the long distance-transportation impact [7]. Furthermore, the energy consumption for the preparation of earth-based materials is approximately 1% of that needed to produce traditional building materials [8,9]. The earth mortar preparation is also less energy-intensive than traditional cement-lime mortars [10].

Moreover, raw earth is a natural material, recyclable, non-toxic, and, thanks to the high hygroscopicity, it can act a moisture-buffering effect, balancing indoor humidity and promoting healthy living conditions in indoor environments [7,11]. 

Although raw earth presents several ecological advantages and is among the most widely used materials, its vulnerability against water is among its weakest features. The collapsing phenomenon in soil is complex and includes fabric, initial moisture content, initial dry density and loading condition [12]. When exposed to a source of water (natural, such as rainfall, or human made) that can enter the soil porous network, collapse may be triggered: after critical moisture content is passed, the fine silt or clay bridges that are providing the cementation in the dry condition will soften, weaken or dissolve, and the structure no longer resists. The total porosity [13] and its typical dimension are the parameters that most influence the permeability of the material, with implications on construction practices and weathering performances. 

As raw earth alone may not fully achieve the performance required to a building product [9], such as in the case of compressive strength for dry samples that should be 2–5 MPa [14], recent research papers focused their attention on many admixtures, mineral additions and stabilizers to improve the cohesion, mechanical properties and moisture buffering capacity [15,16,17].

This aspect paves the way to the second main limitation related to unfired earth, which is the high variability of the material caused by the local variations. Therefore, earth must be characterized in detail in order to identify specific actions and ideal additives to enhance performances and fit the requirements for a specific construction technique [18]. 

Stabilizers such as lime, gypsum and cement were considered to improve the durability and mechanical properties of earth-based products [19]. However, in order to preserve the sustainability added value of raw earth-based construction, the choice of natural sources, possibly derived from wasted biomass, for extracting and/or producing bio-chemical stabilizers [20] should be pursued, in order to reduce overall environmental impact and to support future recyclability [15]. 

In this context, among the natural biopolymers, cellulose undoubtedly represents the most abundant, renewable, and biodegradable source, featuring among the most sustainable raw materials on Earth [21]. Moreover, while cotton and wood can be considered the natural reservoir of cellulose, the high and wide availability of wasted biomass, such as agricultural and food waste, and industrial biowaste (tissue production and paper mill discard, wood from building demolition), provides an alternative source of cellulose which meets the virtuous criteria of a circular economy.

Formed by linear macromolecules of *β*-1,4-linked *D*-glucopyranose units, cellulose fibers show a hierarchical structure, which can be cleaved by mechanical, chemical, and/or enzymatic approaches, leading to the formation of nanocellulose (NC), in the form of nanofibers (CNF) and nanocrystals (CNC), characterized by at least one dimension under 100 nm [22]. 

When reduced to the nanometric dimension, cellulose exhibits new characteristic properties, such as a high tensile and mechanical strength and a high surface area and consequent reactivity [23]. Moreover, CNC can be produced industrially at a tons-per-day scale [24], while CNF suppliers are increasing as well and we would expect an increment on large-scale production in the near future. For all these reasons, it is finding application in different fields, ranging from packaging, as an additive to enhance barrier effect, and dry and wet mechanical resistance [25,26], to environmental remediation [27], as a building block in composites for water and air decontamination [28,29,30,31]. Recently, nanocellulose-based materials are even suggested in fields such as energy conversion [32] and functional materials [33].

In the building sector, the influence of NC, and in particular CNF, has also been widely investigated as a modifier of rheology, and for its influence on hydration and mechanical properties (such as elastic modulus, flexural and compressive strengths) of cementitious materials [34,35,36,37] and alkali-activated ground granulated blast-furnace slag [38]. Furthermore, the use of natural fibers as reinforcement is quite an established topic [39]. However, the application of nanocellulose in earth-based materials is still in its infancy: Stanislas et al. has examined the effect of cellulose [40,41] and of nanofibrillated cellulose [42] on extruded cement-stabilized earth-based matrices; Takahashi et al. [43] studied the influence of cellulose nanofibers on cement-stabilized soils. Finally, the highly hydrophilic character of NC, due to the presence of a large number of alcohol groups, on the one hand, could increase the cohesion of earth plasters by limiting their porosity and, on the other hand, could promote higher water retention.

Therefore, the present paper is focused on the preparation and characterization of different earth plasters by the addition of CNF, derived from both virgin and recycled sources, to two raw earth samples, in order to define the role of such CNF in modifying the adhesion and absorption properties.

Among the protocols available for the production of CNF, 2,2,6,6 tetramethyl-1-piperidinyloxy (TEMPO)-mediated oxidation in the presence of NaBr/NaClO was selected [44]. This approach is highly competitive, sustainable, and scalable compared to other processes [45,46] and also limits depolymerization effects. Moreover, to the best of the authors’ knowledge, this is the first time that CNF obtained and modified following this oxidative procedure are used as additives for earth plasters.

## 2. Experimental

**Scheme 1:**
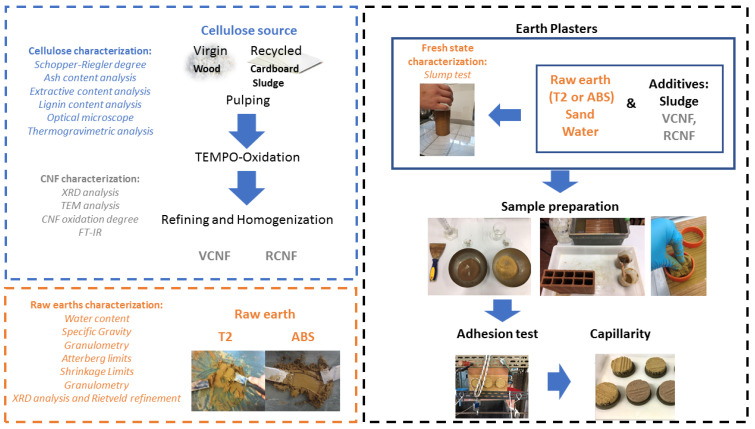
Schematic description of the experimental procedure.

A brief description of how the experimental part will be structured is given in Scheme 1.

### 2.1. Materials

#### 2.1.1. Cellulose-Based Additives

##### Cellulose Sources

To produce cellulose nanofibers, lignocellulosic biomass derived from virgin and waste industrial sources were selected. 

Among virgin sources, spruce fibers (V-wood, provided by Bartoli paper factory, Capannori, Lucca, Italy) were chosen. These fibers, derived from bleached spruce kraft virgin pulp, have a medium length between 1 and 4 mm. The waste sources considered are recycled cardboard (R-pulp, provided by Innovhub SSI, Milano, Italy) and a paper mill sludge (R-sludge, provided by Innovhub SSI, Milano, Italy) derived from the mechanical cleaning stage of a tissue mill factory. Both sources have a good content of cellulose: 65% for recycled pulp and approximately 70% for paper mill sludge (see Section 3.1). The fibers of the latter have a significantly shorter length compared to V-wood and R-pulp (Table 1).

##### Preliminary Cellulose Sources Characterization

All the reagents were purchased from Merck (Darmstadt, Germany). Standard ISO tests were performed using a Sonorex Super RK 512 H ultrasound bath by Bandelin Electronic (Berlin, Germany), a FVG2 autoclave by Fedegari Group (Albuzzano, Italy), a behr Labor-Technik™ (Düsseldorf, Germany) behrotest™ R 104 S Soxhlet Extractor Unit and a Schopper–Riegler Freenes Tester by FRANK-PTI GmbH (Birkenau, Germany). 

Cellulose sources were characterized by analyzing several aspects. The Schopper–Riegler degree (°SR) for each source was performed according to [47]. Ash content was determined following procedure in [48]. Extractive content analysis was performed according to [49]. Finally, lignin content was determined following method in [50]. 

Optical microscope analysis was recorded using an Olympus (Tokyo, Japan) BX-51 optical microscope, with 10× magnification and using the Graff C iodine-based reagent. TGA analyses were performed with a Perkin Elmer (Waltham, MA, USA) STA6000 instrument, in air and N_2_, in the temperature range 30–900 °C, with 10 °C/min scanning.

##### CNF Production Processes

All the reagents were purchased from Merck (Darmstadt, Germany). Deionized water was produced with a Millipore Elix^®^ Deionizer with Progard^®^ S2 ion exchange resins (Merck KGaA, Darmstadt, Germany). Other pieces of equipment used in the procedures include a Branson SFX250 Sonicator (Emerson Electric Co., Ferguson, MI, USA) equipped with a 6.5 mm probe tip, a GEA Niro Soavi (Parma, Italy) NS3006 Land high-pressure homogenizer and a PFI (Trondheim, Norway) refiner compliant with ISO standards [51]. 

TOCNF were obtained through the combination of TEMPO-mediated oxidative pre-treatment followed by high-pressure homogenization. In the following, they will be defined as TOHO. 

For the synthesis of TOHO, the first step was TEMPO-mediated oxidation according to a procedure previously reported in the literature [44,46,52]. Briefly, the selected cellulose source (100 g) was suspended in deionized water (2 L) at a concentration of 5% *w/w* by means of a pulper. This is necessary for V-wood and R-pulp sources, since they are in a pressed sheet form. R-sludge, on the other hand, is already in a suitable form for direct addition to the reaction batch without dispersion through the pulper. Simultaneously, TEMPO (2.15 g) and KBr (15.42 g) were put in a 10 L keg and dissolved in 2 L of deionized water. Once the paper was homogeneously pulped in water, the suspension was transferred in the keg and water was added in order to obtain a final concentration of 2% *w*/*w* (5.7 L as total amount of deionized water). While keeping the solution stirred, a pH meter and two dropping funnels were installed above the keg, with one of the funnels containing an aqueous solution of NaClO (12% *w/v*, 437 mL) and the other a NaOH 4 M aqueous solution (10 mL). NaClO was then slowly dripped into the cellulose suspension and pH was monitored to maintain pH at above 10.5–11 by dripping NaOH 4 M; then, the solution was left stirring overnight. After 12–16 h, the oxidized cellulose was acidified with HCl 12 M (10 mL) to promote aggregation of the cellulose fibers and their easy recovery from water. The oxidized cellulose was then filtered on a Büchner funnel equipped with a tissue filter and washed with deionized water up to neutrality. Reaction yields will be discussed in detail in Section 3.2. The material was then resuspended in deionized water at a 10% *w/w* concentration for the refining phase [53], through which the cellulosic suspension was refined up to a SR degree of 75–80 (maximum limit of cellulose refining that can be obtained with laboratory instruments). Additionally, in this case, the PFI instrument was used, reaching a °SR of 75–80 with 5000 rotor revolutions of the refiner. After the refining treatment, the cellulose pulp underwent homogenization [54]. Each sample was prepared in an aqueous suspension of 6 L at a fiber concentration of 2% *w/w*, maintained at room temperature. The homogenizaton cycles were conducted at different operating pressures, specifically 1 cycle at 500 bar, 1 cycle at 1000 bar, 2 cycles at 1300 bar and 3 cycles at 1500 bar, for a total of 7 discontinuous cycles, followed by a final recovery of the 2% *w/w* non-oxidized nanocellulose suspension.

Powder XRD patterns were recorded on a Brüker (Billerica, MA, USA) D2 Phaser X-ray powder diffractometer using CuKα radiation. The data were measured in the 2θ range 4°–30° with a step size of 0.02° and a counting time of 2 s per step. The data were recorded at room temperature using the Bragg–Brentano reflection geometry.

The crystallinity index (CrI) was determined by the Segal method [55] as shown in Equation (1) and used by others [56]:

(1) $$\mathrm{CrI}=\frac{\left(I_{200}-I_{\mathrm{am}}\right)}{I_{200}}*100\%$$

where CrI is the crystallinity index (%), I_200_ the maximum intensity of the (200) reflection, at 2θ of 22°–23°, and I_am_ is the lowest intensity of diffraction at 2θ of 18°–19° for the amorphous part.

The morphology of cellulose-based additives was determined by TEM (Transmission Electron Microscope Philips CM 200, Koninklijke Philips N.V., Amsterdam, The Netherlands) analysis, with an acceleration voltage of 200 kV, using a pure C film as a grid.

Calculation of the oxidation degree of CNF was estimated as the mmol of carboxylic groups per gram of CNF, by dispersing 500 mg of CNF in deionized water (15 mL), ultrasonicating, and the titrating final dispersion with NaOH_aq_ 0.01 M, using phenolphthalein as a colorimetric indicator (one drop of 10 mM solution in acetonitrile). 

The concentration of the carboxyl groups was calculated by means of the following equation (Equation (2)):

(2) $$\left[\mathrm{COOH}\right]=\frac{M_{\mathrm{NaOH},\mathrm{sol}}{*\;x}_{\mathrm{COOH}}{*\;V}_{\mathrm{NaOH},\mathrm{sol}}}{m_{\mathrm{TOCNF}}}\;$$

where $M_{\mathrm{NaOH},\mathrm{sol}}$ is the moles of NaOH in 1 L of solution, $x_{\mathrm{COOH}}$ is the molar fraction of COOH per mol of NaOH, $V_{\mathrm{NaOH},\mathrm{sol}}$ is the dripped volume of NaOH solution and $m_{\mathrm{TOCNF}}$ is the titrated mass of the additive. 

The solid-phase FT-IR spectra of the samples were obtained using a Varian 640-IR spectrometer (Palo Alto, CA, USA) equipped with a single-bounce ZnSe ATR accessory. All the samples were treated with HCl 0.1 N before spectra acquisition. 

#### 2.1.2. Raw Earths

Two different raw earths were used in this study: the first, called T2, was provided by Industrie cotto Possagno (TV), the second one, ABS, was supplied by Minerali Industriali S.r.l (Lozzolo, Italy). The grain size distribution was obtained combining different methods for the characterization for the coarse and the fine fractions as indicated in [57] using mechanical sieving for grains with a diameter between 2 and 0.075 mm and hydrometer analysis for the finer ones (<0.075 mm), which is based on the velocity at which soil grains of different diameter settle out of a suspension from liquid, after the addition of hexametaphosphate as deflocculating agent.

Atterberg limits were determined as reported in the European standard [58].

Mineralogical characterization of the two samples was performed by means of X-ray powder diffraction, using a Brüker (Billerica, MA, USA) D8 Advance diffractometer with a graphite-monochromator and Cu-Kα radiation. Qualitative phase composition was analyzed with the open-source software Profex [59,60] (Figures S1 and S2 in Supporting Information). Quantitative mineralogical phase composition was determined through Rietveld refinement with the code GSAS [61]. A 10 wt% of ZnO was added to all the analyzed samples as an internal standard to quantify the crystalline and the amorphous fractions [62]. 

### 2.2. Sample Preparation

The cellulose-based additives used in the raw earth mixtures are listed below: 

Untreated paper mill sludge (R-sludge), TOHO from spruce (VCNF-wood), TOHO from recycled paper pulp (RCNF-pulp) and TOHO from paper mill sludge (RCNF-sludge). 

The earth, sand and admixtures were hand mixed, gradually adding small quantities of water to obtain a good level of plasticity, a necessary prerequisite for the homogeneity of the samples. The adopted reference compositions were reported in Table 2: the mixtures were prepared using an earth: sand ratio of 1:4 for T2 earth, and 1:3 for ABS earth, and the additives were added at 1% *w/w* of the dry weight of the sample.

The optimal water content, to ensure comparable workability across different mixtures, was determined through a slump test. For this test, the mixture was pressed into a cylinder up to a height at least twice the diameter (7 cm diameter and 14 cm height); afterwards, it was slowly lifted vertically to check if the mixture was self-standing, i.e., the diameter and height did not change. If the mixture was too dry (cracks were visible in the cylinder) or too moist (the cylinder shape was not maintained), the water content was changed accordingly, and the test was repeated.

### 2.3. Adhesion Tests

The test [63] to determine the adhesive strength of hardened plaster on substrates requires a fired brick tile (25 × 25 cm^2^ with a thickness of 1.5 cm) as support. To avoid contact imperfections, the bricks were first submerged into the water for 10 min, then a slip (a liquid mixture of water and soil) was brushed on the surface. The formwork is a plastic ring with diameter of 5.8 cm and height of 1.5 cm. 

The formworks were filled uniformly and completely, and the surface was levelled; at the same time, a portion of the obtained mixture was put in an oven at 105 °C for 24 h to evaluate the exact water content [64]. An additional sample was then prepared for moisture controlling purposes and left in the same room together with the samples for the adhesion test, until the water content of the control sample measured with a soil-hygrometer decreases to below 1%. 

After the first week in a controlled environment (T = 20.2 ± 0.8 °C and RH = 55 ± 6.0%), if the samples were still moist, they were put in an oven at 40 °C for a few hours in order to attain the correct water content.

**Figure 1:**
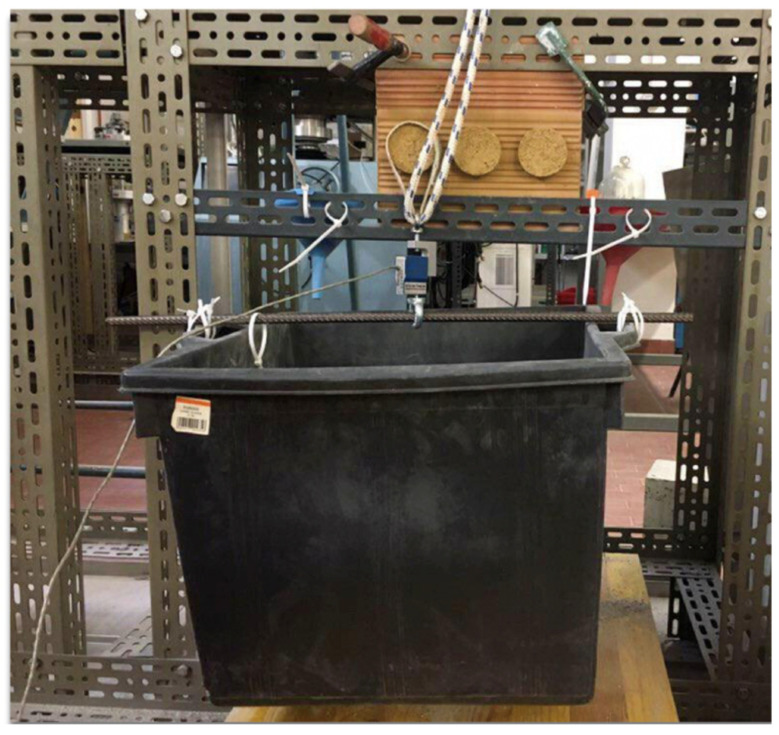
Experimental set up for adhesion tests.

The brick was fixed onto a vertical grid by means of clamps. An empty container was secured onto the sample with a resistant harness and connected to a load cell used to continuously measure the load applied on the sample. The container was progressively filled with sand unit its weight was able to remove the sample from the surface, as shown in Figure 1. Four samples for each mixture were prepared to extrapolate (averaging and cross-checking) a more reliable result.

### 2.4. Capillary Water Absorption

The capillary water absorption test is useful to find the maximum amount of water that can be absorbed in comparison to the volume or mass of the sample, known as “capillary water capacity” (kg/m^3^). This is an important value when considering the condensation phenomena in building components.

The procedure is based on the standard in [65] with adaptation to earthen mortars. The necessity to adapt capillary water tests to earth-based materials is common [66] due to high vulnerability to liquid water of the analyzed material. The bedding layer was replaced with a high-permeability rigid porous stone, also substituting the metallic mesh proposed in other research works [66,67], which has the same permeability properties of those suggested in the reference standard and continues to guarantee a uniform distribution of water on specimen bottom surface. The porous stone also gives the possibility to significantly reduce both soil loss at the soil–water interface and specimen damage risk when moving for weighting. A further improvement was introduced to avoid any high-temperature-induced porous structure change, which can occur in soil specimen: oven drying was replaced with air drying at laboratory room temperature and relative humidity (temperature between 20 and 23 °C and relative humidity 40–50%) until constant dry weight is reached. The residual hygroscopic water content (estimated to be approximately 3% for loose soil) can be assumed not to affect the capillary rise. 

**Figure 2:**
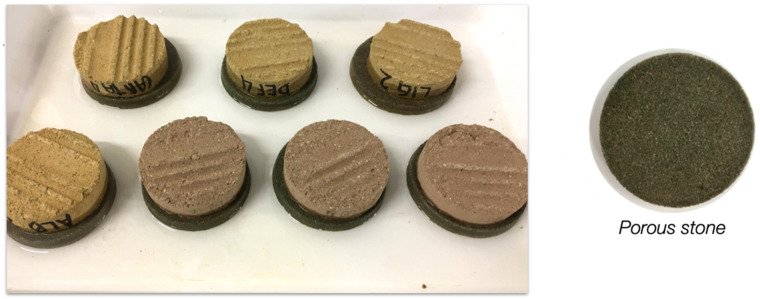
Experimental set up for capillary absorption tests.

The capillary absorption test was performed on the specimens used in the adhesion test, once detached from the brick and placed on a saturated porous stone, which was previously saturated in water by immersing for 24 h, as shown in Figure 2. The weight of the saturated porous stone is also recorded before placing soil disc. After the weight of the specimen and its support porous stone was recorded regularly at constant time interval, the amount of water P_w_ retained by the soil is evaluated by subtracting (soil + porous stone) the weight of the saturated porous stone, which can be assumed to be constant through the entire test, from the gross weight.

In this way, during the calculation of the amount of water retained by the specimens, the precise value of the saturated porous stone, which is given by the weight of the porous stone itself plus the retained water (Equation (3)), was subtracted:

(3) $$P_{w}={\;P}_{\mathrm{tot}}-\left(P_{\mathrm{ps}}+{\;P}_{d}\right)$$

where P_tot_ is the weight of the sample on the porous stone, P_ps_ is the weight of the saturated porous stone, and P_d_ is the weight of the dry sample measured before the beginning of the test. The ratio between P_w_ and the cross-sectional area of the specimen will lead to the absorbed water per unit area value.

## 3. Results and Discussion

### 3.1. Cellulose-Based Additives Characterization 

As reported in the Experimental Section (Table 1), three different cellulose sources were considered and characterized for the production of TOHO CNF: a virgin one (V-wood) and two derived from waste sources—more specifically a recycled cardboard (R-pulp) and a paper mill sludge derived from the mechanical cleaning stage of a tissue mill factory (R-sludge). 

The composition of the three sources was determined and is reported in Table 3. As expected, V-wood had an almost exclusive content of holocellulose (cellulose and possible hemicellulose), which decreased to approximately 70% for the two recycled sources. The remaining 30% was a balance of ash and lignin content in R-pulp, while there was much more lignin (23%) in R-sludge. These results were confirmed by TGA (Figures S3 and S4 in SI). Indeed, residuals at 900 °C, which mainly depend on ash content, were approximately 1.4%, 3.5%, and 12.0% for V-wood, R-sludge, and R-pulp, respectively. 

The cellulose pulps were also characterized by the Schopper–Riegler, (°SR) degree. The R-sludge showed a slightly higher °SR value than the other two sources, which suggests a higher fibrillation of the cellulose fibers with increased surface available for chemical oxidation (Table S1 in SI).

**Figure 3:**
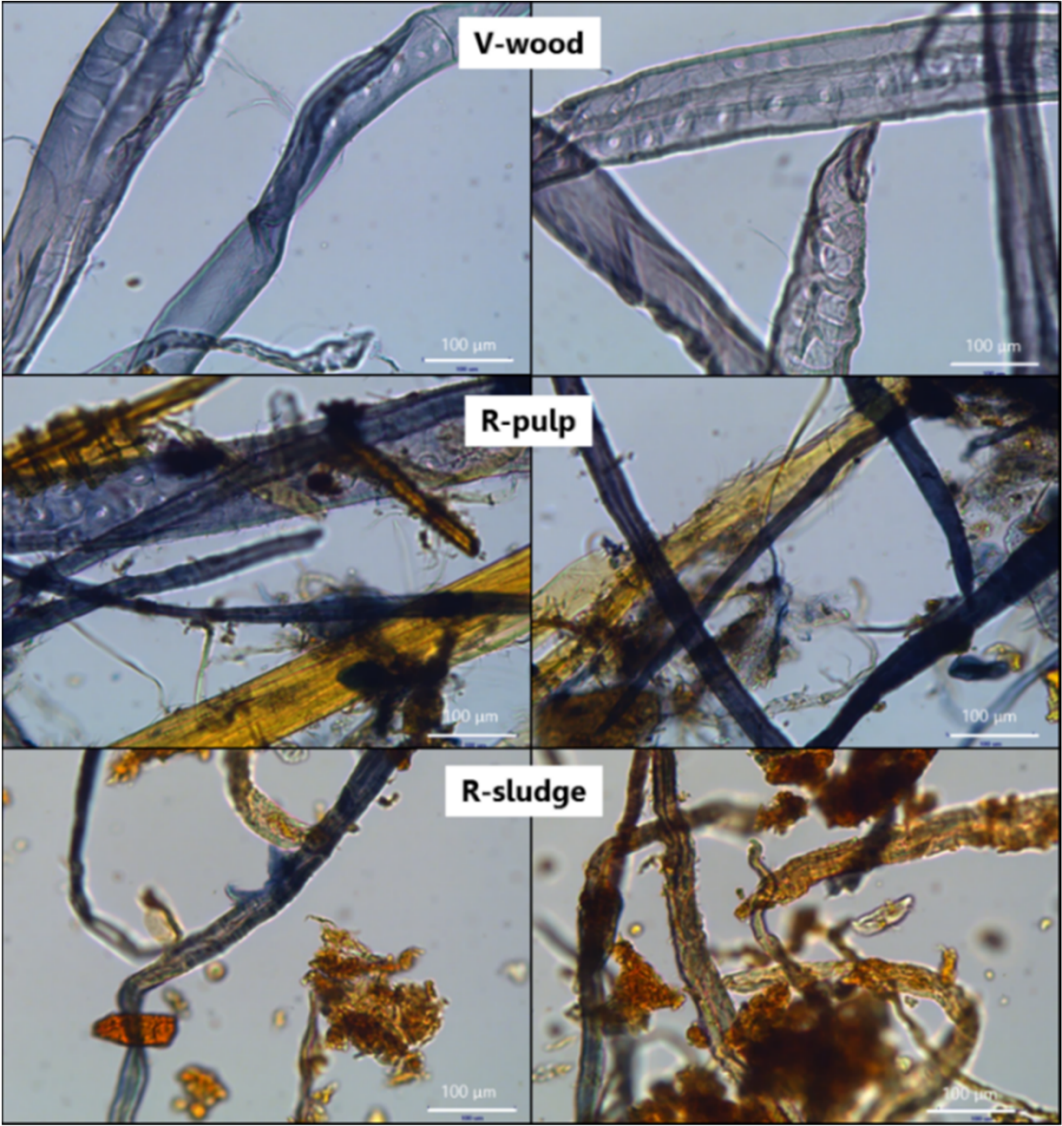
Optical microscope images of the three sources used to produce the additives.

This result was somehow confirmed by optical microscope analysis (Figure 3). The sample V-wood consisted of fibers of coniferous softwood bleached chemical pulp. There were only traces of bleached hardwood chemical pulp of broadleaf trees (<2%). Fibers were homogeneous in diameter, which was in the order of tens of µm. R-pulp was characterized by an inhomogeneous distribution of fibers, consisting of conifer and broadleaf hardwood mechanical pulp, conifer and broadleaf bleached chemical pulp, and conifer and broadleaf unbleached sulfate chemical pulp. The bleached chemical pulp consisted of conifer fibers and broadleaf fibers in similar amounts to each other, while the mechanical pulp was made up of conifer fibers and broadleaf fibers, with a prevalent coniferous component. Finally, the unbleached sulfate chemical pulp consisted of conifer fibers and broadleaf fibers, with a slightly prevalent coniferous component. Overall, the sample showed a fair amount of mechanical pulp. Finally, R-sludge showed fibers of different nature, difficult to identify because they appeared broken and dirty. However, bleached chemical fibers were clearly the major component although it was not possible to clearly determine its origin (hardwood or softwood).

All three sources underwent TEMPO-mediated oxidation, according to the reported procedure, in order to produce the corresponding TOHO CNF. This approach consists of the regioselective conversion of the primary alcohol groups on the C6 position of the glucopyranose unit to the corresponding carboxylic moieties. Moving to an alkaline medium, which promotes deprotonation of carboxylic groups, it is possible to achieve single-TEMPO-oxidized cellulose nanofibers by taking advantage of the electrostatic repulsion among negatively charged nanofibers. This nanodefibrillation is assisted by high-pressure homogenization. In all the cases, yields of the oxidation processes were almost quantitative (Table 4), if referring to the determined holocellulose content as reported in Table 3. By considering the oxidative process in the presence of sodium hypochlorite, and the subsequent alkaline conditions to promote CNF defibrillation, it is also reasonable to conclude that the hemicellulose content of original sources was in all cases negligible.

Unfortunately, TGA results are unable to clearly decouple cellulose and hemicellulose thermal decomposition ranges. Poletto [68] states that the degradation of hemicellulose takes place at approximately 300 °C, and the main degradation of cellulose occurs at approximately 350 °C. However, it is difficult to quantify with certainty their relative amounts from the analysis of thermograms [69]. In any case, the thermal behavior of hemicellulose has large variations depending on the origin of the hemicellulose itself [70]. This is even more difficult in samples with a high amount of lignin, which is known to be very stable and more difficult to decompose than cellulose or hemicellulose; moreover, lignin decomposes slowly over a large temperature range, approximately from 250 to 600 °C [71]. The second DTG peak approximately 716 °C of R-pulp accounts for the high ash content; indeed, no such peak is present in the other two samples whose ash content is negligible or very low (Figures S3 and S4 in SI). TGA of CNF confirms the main holocellulose decomposition phenomenon with a DTG peak approximately 350 °C and the disappearance of the high decomposition peak in R-pulp, suggesting a purification of the material. However, the thermal behavior of CNF from R-sludge is somewhat different from the others oxidized samples, with a larger residual mass at 850 °C. As a confirmation of these findings, TGA were also performed in air (Figure S3 in SI). Thermograms are somewhat more complex than in nitrogen flow, mainly showing a split of the main DTG peak, suggesting a different decomposition onset of cellulose and hemicellulose. 

The expected conversion of C6 alcohol groups to the corresponding carboxylic groups was confirmed by FT-IR analysis, which showed the appearance of the carboxylic C=O stretching band at 1722 cm^−1^ for all the TOHO CNF samples (Figures S5–S7 in SI). The oxidation rate, which is expressed in mmol of carboxylic groups per gram of TOHO CNF for all the samples, is also reported in Table 4. Surprisingly, this value is double for RCNF-sludge compared to the VCNF-wood, probably due to the smaller fibers present in R-sludge, and the consequently higher exposed surface area.

**Figure 4:**
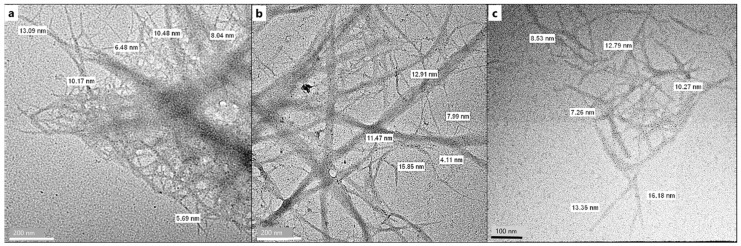
TEM images of cellulose-based additives obtained by the TOHO process. (**a**) VCNF-wood; (**b**) RCNF-pulp; (**c**) RCNF-sludge.

The formation of TOHO CNF was confirmed by TEM analysis (Figure 4). While for all the samples it was possible to obtain evidence of the nanometric diameter of the fibers, TEM images clearly show the smaller dimensions of RCNF-sludge.

Finally, the crystallinity indexes of all materials (Table S2 in SI) were determined by XRD, as reported in the Experimental Section. The diffractograms are shown in the SI (Figure S8 in SI). It can be observed that the crystallinity indexes are all very similar and lie in a range between 60 and 70%. A slight decrease in the CrI is observed in all samples after TOHO treatment. This is also supported by TGA, where thermal decomposition onset occurs at a lower temperature in RCNF-pulp and RCNF-sludge samples (Figures S1 and S2 in SI), while the shift is unclear for VCNF-wood. Indeed, thermal decomposition is shifted to lower temperatures with decreasing cellulose crystallinity and crystallite size [68].

### 3.2. Raw Earths Characterization

In order to consider the common variability of raw earths and to evaluate the effect of TOHO additives compared to the different earth compositions, two reference soils were considered for application tests, namely T2 and ABS.

**Figure 5:**
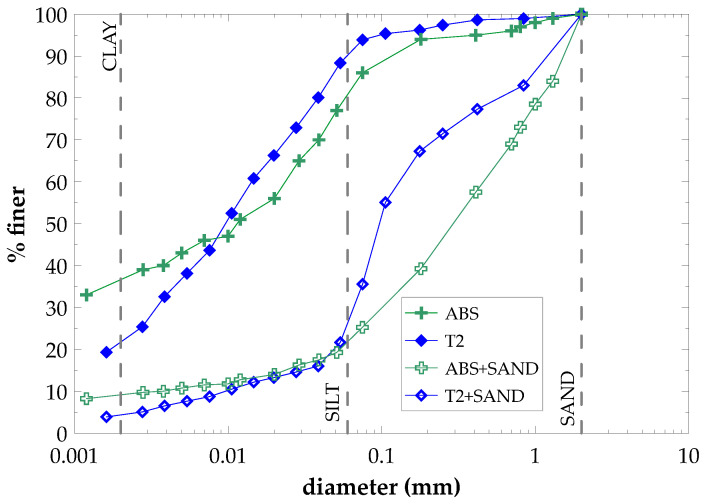
Grain size distribution for reference soil and plaster mix.

Soil properties have been evaluated according to the test methods previously introduced, namely specific gravity, grain size distribution and Atterberg limits. A summary is reported in Table 5, providing the different soil fraction percentage estimated from grain size distribution with detailed representation (sieving and hydrometer analysis [72]) shown in Figure 5. 

**Figure 6:**
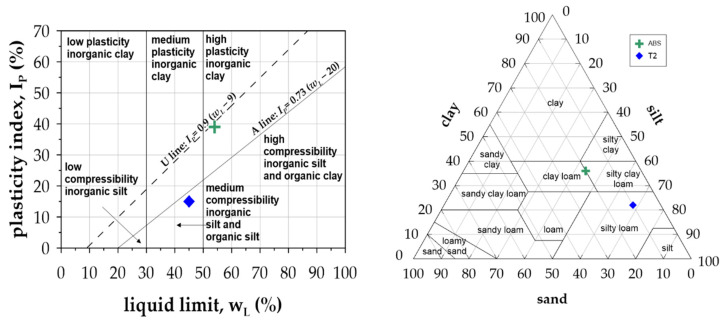
Casagrande plasticity chart and USDA texture triangle.

According to the USDA texture triangle, T2 can be classified as a silty clayey loam, while ABS as a clayey and silty loam. Regarding the Atterberg limits, based on the Casagrande plasticity chart, T2 is defined as a high-plasticity clay and ABS as a medium-compressibility silt with no organic matter (Figure 6). 

Grain size distribution shoed that the reference T2 and ABS are different in grain size distribution, especially for the finer fraction (clay), which is higher in ABS soil. Sand addition, in its turn, reduces the differences from ABS and T2 in the finer range, while a slight difference remains in the coarse diameter range. The difference in clay content also seems to affect plasticity behavior, as can be observed in the Casagrande chart report, and again, the USDA classification identifies both soil as loam, underlining the major differences in the clay amount.

The mineralogical composition of the two raw earths is summarized in Table 6. The main clay minerals present in both the earth samples are smectite and illite. These crystalline compounds show opposite behavior in presence of water: only the former is associated with high volumetric changes in presence of water; however, the large amount of quartz may contribute to limit such expansion. Chlorite and kaolinite were also detected in both the materials as well as microcline and albite. 

### 3.3. Earth-Based Plasters 

#### 3.3.1. The Adhesion Test

**Figure 7:**
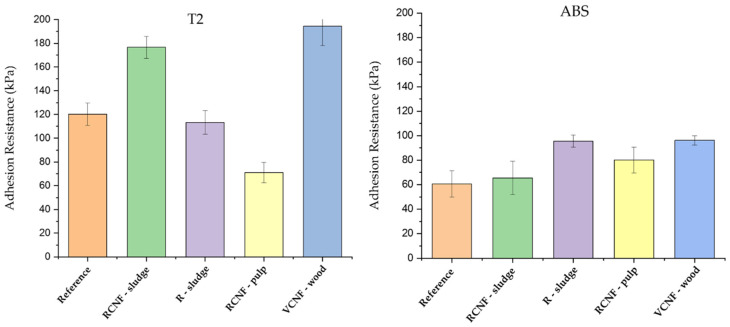
Results of the adhesion test on T2 and ABS earths, using cellulose-based additives.

The results of the adhesion test are shown in Figure 7. In addition to TOHO CNF samples, R-sludge was also directly used. The values obtained with samples prepared with T2 earth show beneficial effects only in presence of RCNF-sludge and VCNF-wood with a concentration 1% wt/wt. The sample with VCNF-wood has adhesion strength, while no visible effect can be observed for samples prepared with R-sludge. Only the sample prepared with RCNF-pulp has lower adhesion strength compared to the reference.

A different trend can be observed for samples prepared with ABS (Figure 7). The samples prepared with R-sludge and VCNF-wood, 1% wt/wt, show higher values of adhesion compared to the reference. Additionally, in these series, the best performance is obtained by the use of nanocellulose from a virgin source, VCNF-wood. Moreover, the sample prepared with R-sludge 1% wt/wt shows an adhesion strength similar to that one obtained with nanocellulose from a virgin source—96.1 kPa and 95.6 kPa, respectively. A slight difference can be observed for the sample with nanocellulose from recycled cardboard (RCNF-pulp) 1% wt/wt. The effect of NC from recycled sludge (RCNF-sludge) 1% wt/wt is negligible.

These results can be due to different clay–additive interactions, types of clay minerals, proportions of soil components and earth–sand ratios.

It has to also be noted that sample preparation is unavoidably affected by the variability of a manual procedure, which, even if accurately controlled at each step (soil and additive in mixture, the amount of water added, dimension checks, etc.). Therefore, the final sample can have some differences, which, in same condition, can lead to a bigger effect in terms of observed mechanical properties, but, in its turn, enables identification of a reasonable range within which it is to be expected that the observed mechanical properties may vary. In adhesion tests, for example, considering that the adhesion between the brick and the earth disk is mostly due to surface interactions, such differences may be due to an even smaller difference in applied pressure when filling the plastic mold. The adhesion results of ABS seem to have a limited beneficial effect from the addition of NC; moreover, the best result is comparable with that of recycled sludge, i.e., a source of cellulose. However, in the case of T2, the use of NC as an additive improves the adhesion performance of the plaster in two out of three cases.

In the literature, we also found that mineral stabilizers, such as cement or lime, were used [42,73,74]. Lima and Faria studied the influence of the addition of only two types of natural fibers, oat straw and typha fiber-wool, on earth plasters, finding an improvement in the adhesion to the substrate [7]. 

More recently, Parracha et al. [20] used an innovative bio-product based on cellular extracts of *Escherichia coli*, cultured in lysogeny broth (LB) medium and supplemented with iron, in earth mortar formulation. Apart from a distinctive porous structure, a general decrease in mechanical properties was clearly observed, including in adhesion strength.

#### 3.3.2. Capillary Absorption Test

The results of this test are expressed by the capillary absorption curve, which represents the amount of water absorbed per unit area (kg/m^2^) as a function of the square root of the elapsed time (s^0.5^). The slope of the most representative initial linear segment of this curve corresponds to the capillary absorption coefficient (CC). The asymptotic value of the curve is an approximation of the total amount of water absorbed by the samples [74].

**Figure 8:**
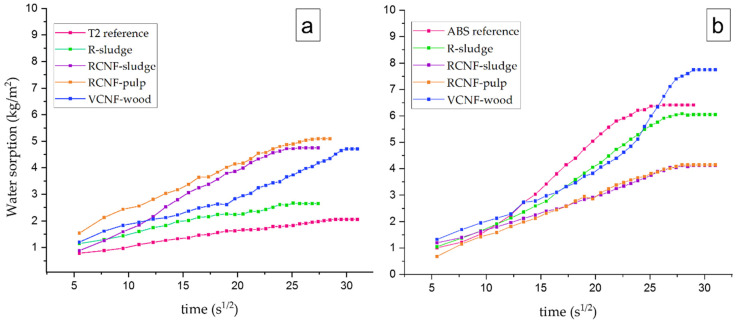
Results of the capillary absorption test on (**a**) T2 and (**b**) ABS earth, using cellulose-based additives.

The capillary absorption curves of T2 samples are shown in Figure 8a; the addition of nanocellulose has a negative effect on the rate of absorption of water as well as on the maximum amount of water absorbed. The reference sample shows both the slope with the lowest gradient and the lowest amount of water absorbed. It should be noted that the samples with added nanocellulose. VCNF-wood, RCNF-sludge and RCNF-pulp, absorb a similar amount of water and reach the maximum capillary water capacity but the rate of absorption over time is different, while sample R-sludge shows a behavior similar to the reference.

ABS samples are shown in Figure 8b; the reference sample is able to absorb water faster than the samples with nanocellulose except for the sample VCNF-wood, which absorbs the highest amount of water. The lowest amount of water absorbed over time is attributed to the samples RCNF-pulp and RCNF-sludge. 

It is also interesting to note that the sample with added VCNF-wood follows the same slope as the sample with added REC-PP until approximately 10 min; and further, the slope of the former increases and reaches the maximum.

As expected, cellulose and nanocellulose increase water absorption capacity in T2-based earth plasters; however, the opposite behavior is found with ABS earth, where the water absorption capacity of mortars formulated with cellulose or NC is lower in most cases compared to the reference sample. In [7], the hygroscopic capacity of the plasters is not affected by the addition of the fibers; however, it is to be noted that they tested water vapor sorption–desorption, so no direct comparison with the present study is possible. 

## 4. Conclusions

In this work, preliminary analysis on the performance of earth-based plasters with (nano)cellulose-based additives was performed. In particular, for the first time, TOHO CNF were investigated in this context. Adhesion tests were carried out in order to assess the maximum vertical load the samples can sustain, investigating if the addition of (nano)cellulose-based additives could improve or deteriorate the adhesive strength of the earth. Comparing T2 and ABS earths, T2 showed the highest shear strength and the addition of R-sludge did not significantly improved the adhesive strength of the samples, while the addition of cellulose nanofibers RCNF-sludge and VCNF-wood demonstrated remarkable improvement. Albeit RCNF-pulp deteriorated the adhesive strength. As for ABS, R-sludge, VCNF-wood and RCNF-pulp significantly improved adhesion strength, while no significant change was recorded on addition of RCNF-sludge. 

Secondly, a capillary test was performed to assess the resistance of materials against water and to determine whether addition of additives modifies resistance against water absorption. It was interesting to note that, in the case of T2, all the additives deteriorated the resistance of the mixture against water. In the case of ABS, however, the addition of additives resulted in a reduction in the amount of water adsorbed, thus having a positive impact in terms of water resistance, except in the case of the VCNF-wood sample, for which a worsening effect was observed.

These preliminary tests give us an idea of how cellulose-based additives can impact earth-based mixtures, and the obtained results pave the way for more in-depth experiments, including those aimed at investigating the potential risk that microbial life present in raw earths could promote the degradation of bio-based CNF additives. Further results may show us whether these fully sustainable mixtures can be used as plasters toward an increasingly greener building industry.

## Figures and Tables

**Table 1:** Fiber length of selected cellulose sources for the production of nanofibers.

Cellulose Source	Category	Fiber Length (mm)
V-wood	Virgin	1–4
R-pulp	Waste	1–2
R-sludge	Waste	0.5–1

**Table 2:** Earth plasters composition.

Sample	Earth/Sand Ratio w/w	Water %w/w_dry_	Additive %w/w_dry_
T2 reference	1:4	0.18	-
T2 RCNF-sludge	1:4	0.18	0.1
T2 R-sludge	1:4	0.18	0.1
T2 RCNF-pulp	1:4	0.18	0.1
T2 VCNF-wood	1:4	0.18	0.1
ABS reference	1:3	0.16	-
ABS RCNF-sludge	1:3	0.16	0.1
ABS R-sludge	1:3	0.16	0.1
ABS RCNF-pulp	1:3	0.16	0.1
ABS VCNF-wood	1:3	0.16	0.1

**Table 3:** Summary of the main characteristics of the source materials, including ash, extractives, lignin and holocellulose.

Cellulose-Based Additive	Ash Content [%]	Extractives Content [%]	Lignin Content [%]	Holocellulose Content [%]
V-wood	<1	<1	<1	98
R-pulp	18	1	14	67
R-sludge	3	4	23	70

**Table 4:** Yield for TEMPO-mediated oxidation of cellulose sources and oxidation rate.

Cellulose-Based Additive	Reaction Yield [%]	Oxidation Rate [mmol_COOH_/g_TOCNF_]
VCNF-wood	95	0.498
RCNF-pulp	62	0.330
RCNF-sludge	69	1.004

**Table 5:** Geotechnical parameters for raw earths.

	T2	ABS
Clay (%)	22	36
Silt (%)	68	44
Sand (%)	10	20
d_50_ (mm)	0.009	0.012
d_90_ (mm)	0.052	0.110
Water Content	3.50	3.08
Liquid Limit (%)	54	45
Plastic Limit (%)	15	30
Plasticity index (%)	39	15
Activity Index	1.86	0.42
Shrinkage Limit	9.5	13.7
Specific Gravity	2.77	2.78

**Table 6:** Mineralogical composition of the two earth materials determined by a semi-quantitative analysis.

Earth	Quartz (%)	Smectite (%)	Illite (%)	Kaolin (%)	Chlorite (%)	Microcline (%)	Albite (%)	Amorph. (%)
**T2**	36.9	9.8	15.9	5.4	4.8	4.9	4.9	16.6
**ABS**	32.5	8.9	10.9	-	3.7	5.3	3.1	35.6

## Data Availability

Not applicable.

## Supplementary Materials

The following supporting information can be downloaded at: https://www.mdpi.com/article/10.3390/ma16010074/s1, Figure S1: XRD pattern of T2 earth; Figure S2: XRD pattern of ABS earth; Figure S3: Thermogravimetric analysis of cellulose sources and additives obtained by the TOHO process—analyses performed in air; Figure S4: Thermogravimetric analysis of cellulose sources and additives obtained by TOHO the process—analyses performed in N_2_; Figure S5: FT-IR spectrum of V-wood and VCNF-wood; Figure S6: FT-IR spectrum of R-pulp and RCNF-pulp; Figure S7: FT-IR spectrum of R-sludge and RCNF-sludge; Figure S8: X-ray diffractograms of cellulose sources and additives obtained by the TOHO process; Table S1: Drainage (°SR) of cellulose pulp calculated for each source selected for the production of cellulose nanofibers; Table S2: Crystallinity index of sources and cellulose-based nanodimensioned additives.
