# Supplementary material for: Can TEMPO-Oxidized Cellulose Nanofibers Be Used as Additives in Bio-Based Building Materials? A Preliminary Study on Earth Plasters
Source: Materials (Basel). 2022 Dec 21;16(1):74. doi: 10.3390/ma16010074 (PMC9820849; doi:10.3390/ma16010074)
Supplement: Supplementary file 1 [file materials-16-00074-s001.zip › materials-2068100-supplementary.pdf]

Supporting Information for

# Can TEMPO-oxidized cellulose nanofibers be used as additives in bio-based building materials? A preliminary study on earth-plasters

Paola Gallo Stampino[1,*], Laura Riva[1,*], Marco Caruso[1], Imran Abdul Rahman[1],

Graziano Elegir[2], Daniele Bussini[2], Javier Marti Rujas[1], Giovanni Dotelli[1] and Carlo Punta[1,3]

[1] Politecnico di Milano, Department of Chemistry, Materials and Chemical Engineering "G.Natta", p.zza L. da Vinci 32, 20133 Milano, Italy;

[2] Innovhub Stazioni Sperimentali per l'Industria S.r.l., Via Giuseppe Colombo 83, 20133 Milano, Italy;

[3] Milano Politecnico Research Unit, National Interuniversity Consortium of Materials Science and Technology—INSTM, Via Mancinelli 7, 20131 Milan, Italy.

# Table of Contents

1. Earths XRD……………………………………………………………………………….S2
2. TG analyses……………..…………………………………………………………………S3
3. Schopper-Riegler of cellulose sources…………………………………………………S5
4. FT-IR Spectra……………………………………………………………………………..S5
5. XRD Diffractograms and Crystallinity Index….………..…………………………………S7

# 1. Earths XRD

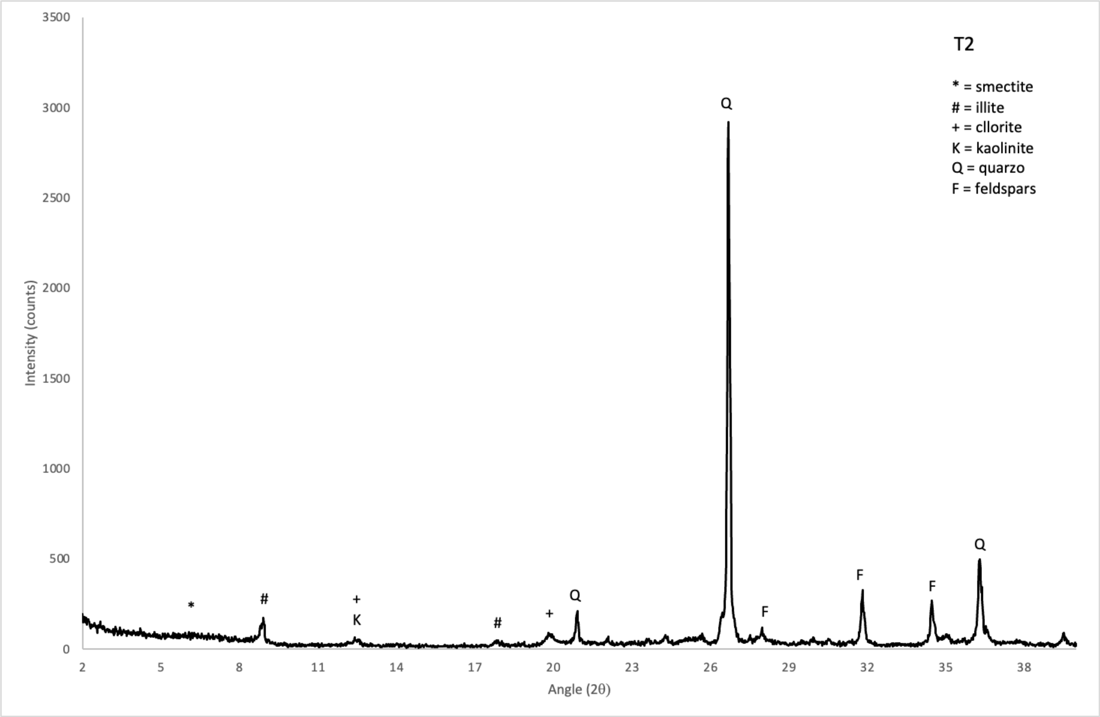


**Figure S1:** XRD pattern of T2 earth.

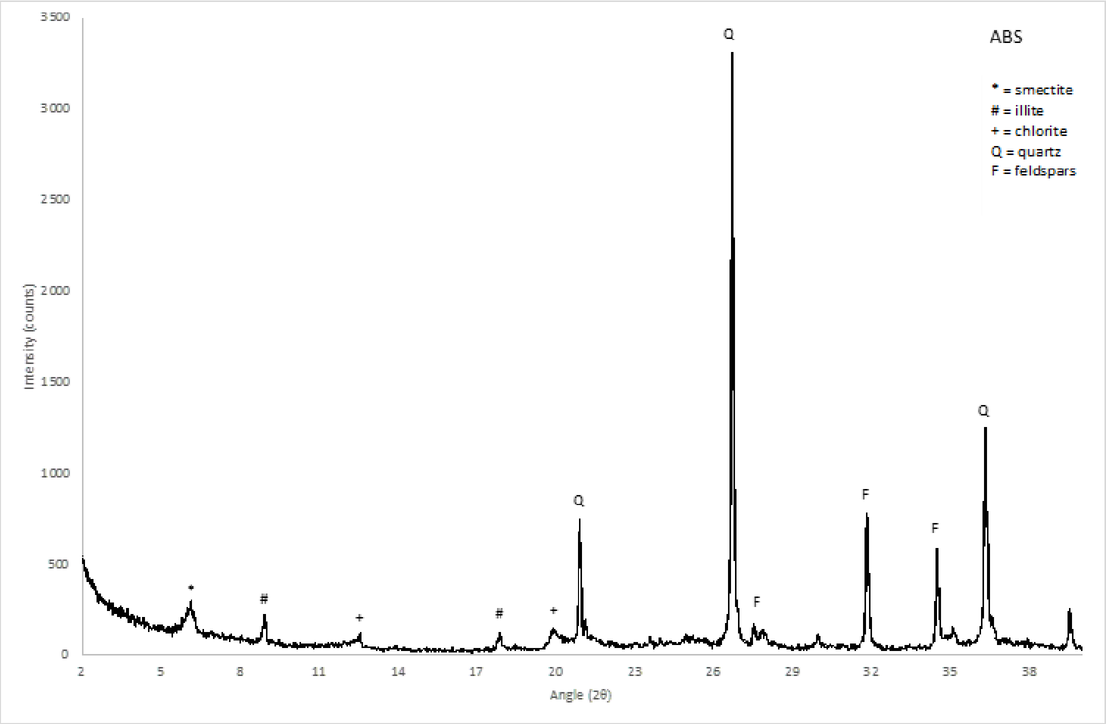


**Figure S2:** XRD pattern of ABS earth.

# 2. TG analyses

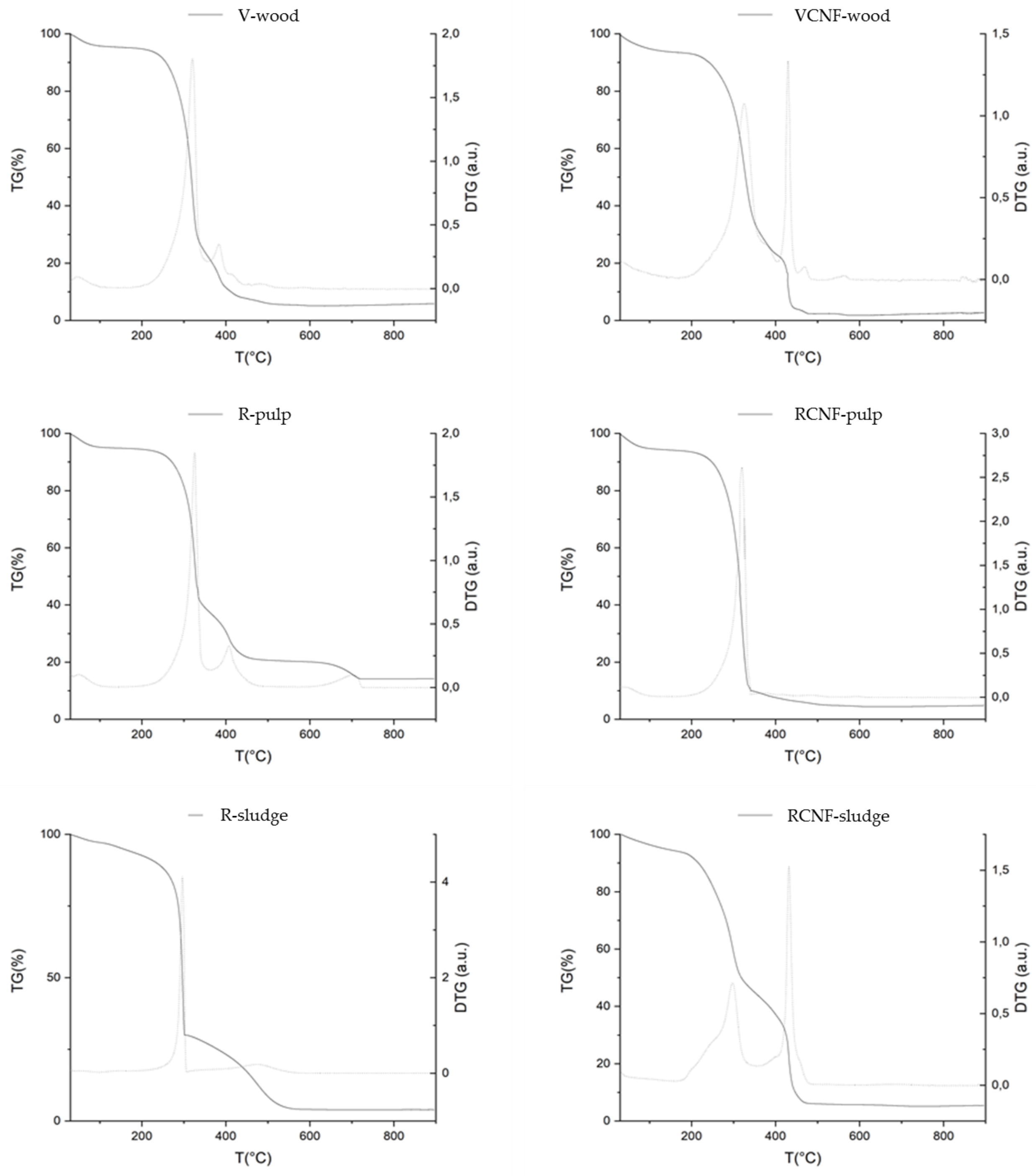


**Figure S3.** Thermogravimetric analysis of cellulose sources and additives obtained by TOHO process; analyses performed in air.

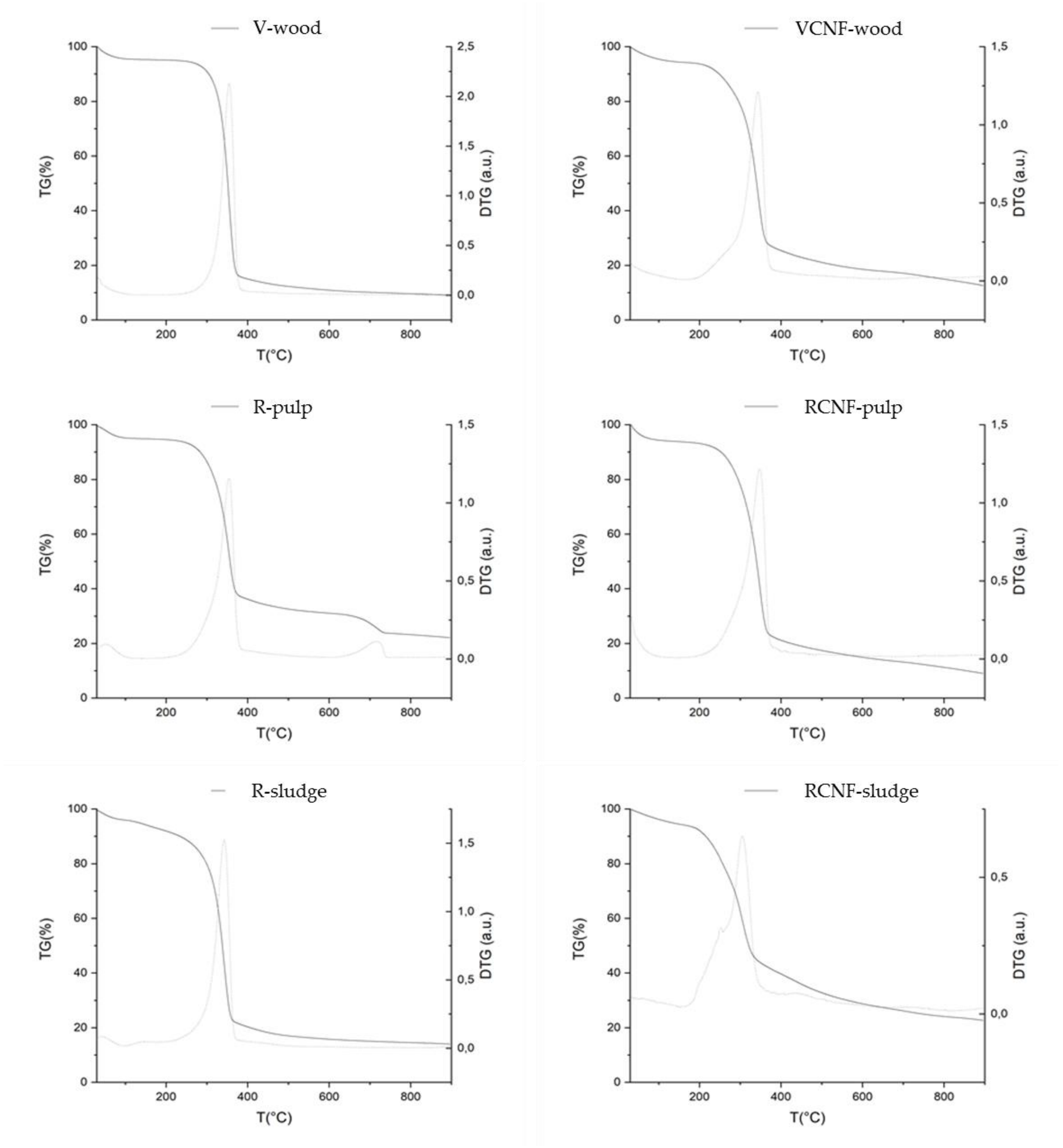


**Figure S4**. Thermogravimetric analysis of cellulose sources and additives obtained by TOHO process; analyses performed in $N_2$.

# 3. Schopper-Riegler of cellulose sources

**Table S1.** Drainage (°SR) of cellulose pulp calculated for each source selected for the production of cellulose nanofibers.

| Source | °SR |
|---|---|
| V-wood | 14 |
| R-pulp | 17 |
| R-sludge | 26 |

# 4. FT-IR Spectra

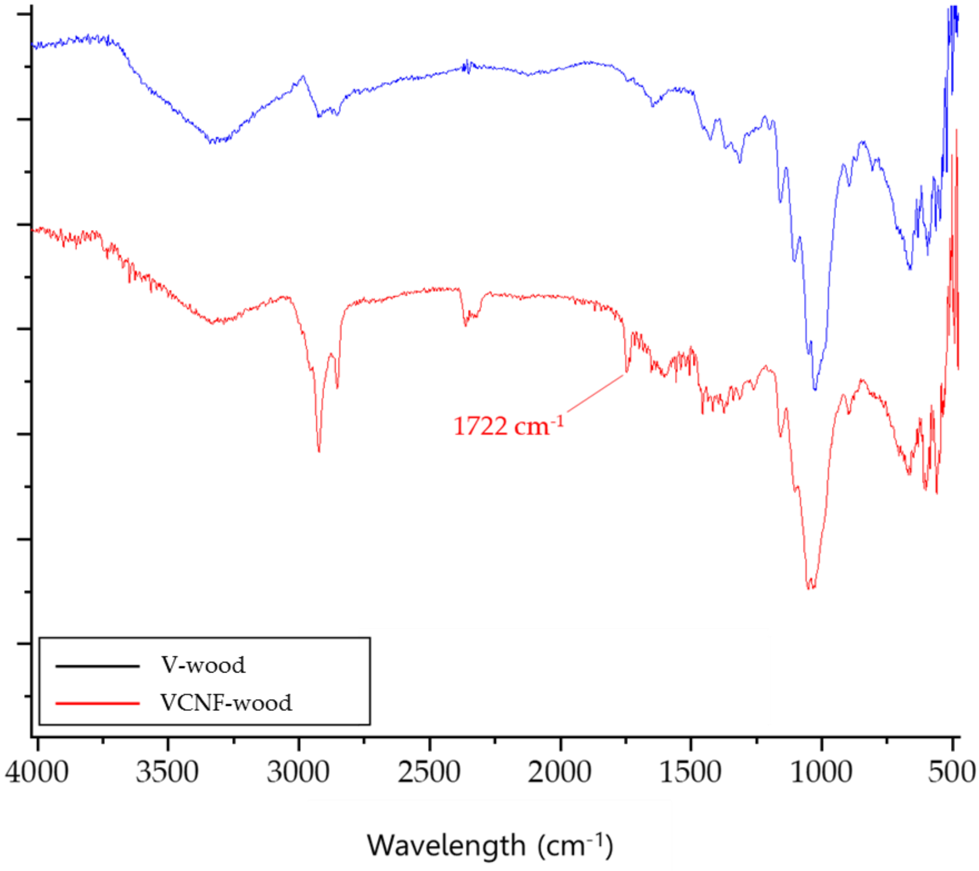


**Figure S5.** FT-IR spectrum of V-wood and VCNF-wood.

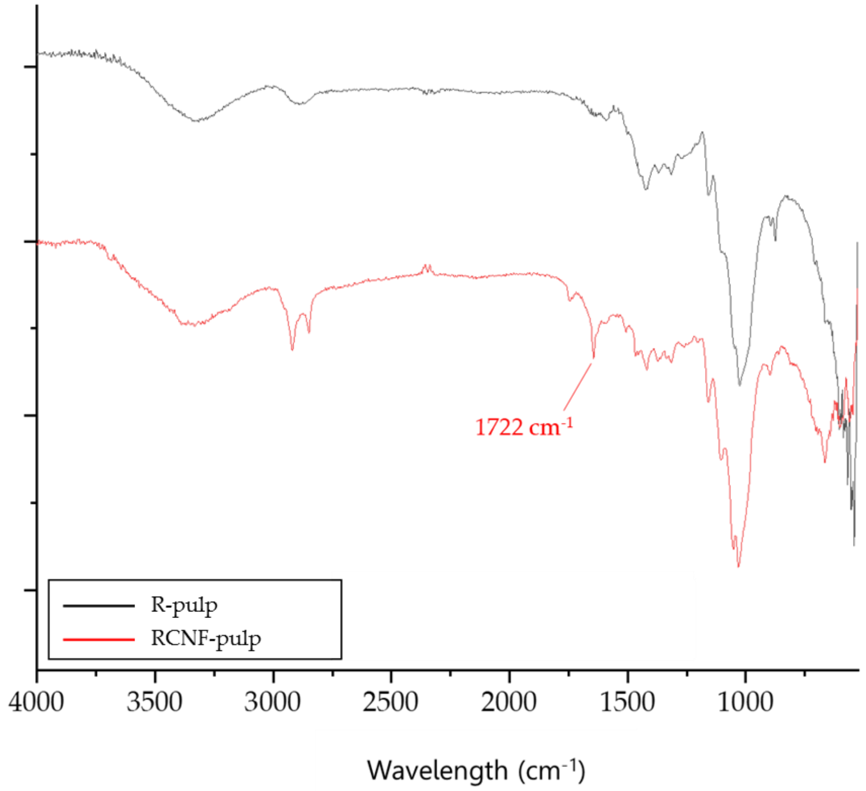


**Figure S6.** FT-IR spectrum of R-pulp and RCNF-pulp.

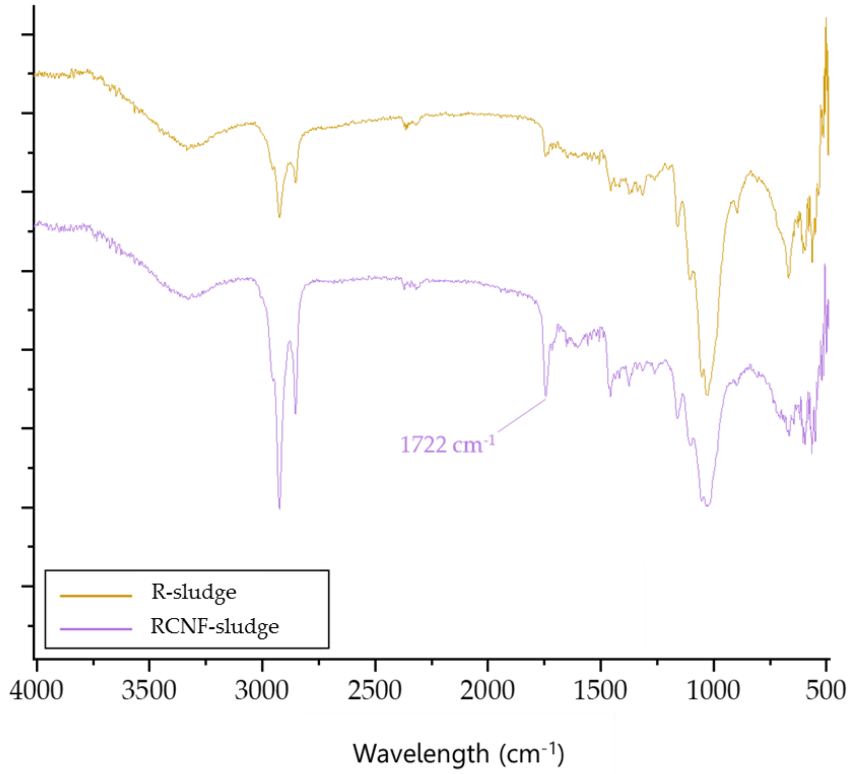


**Figure S7.** FT-IR spectrum of R-sludge and RCNF-sludge.

# 5. XRD Diffractograms and Crystallinity Index

**Table S2**. Crystallinity index of sources and cellulose-based nanodimensioned additives.

| Cellulose-based additive | Crystallinity index [%] |
|---|---|
| V-wood | 68.5 |
| R-pulp | 70.4 |
| R-sludge | 67.5 |
| VCNF-wood | 65.3 |
| RCNF-pulp | 62.2 |
| RCNF-sludge | 67.0 |

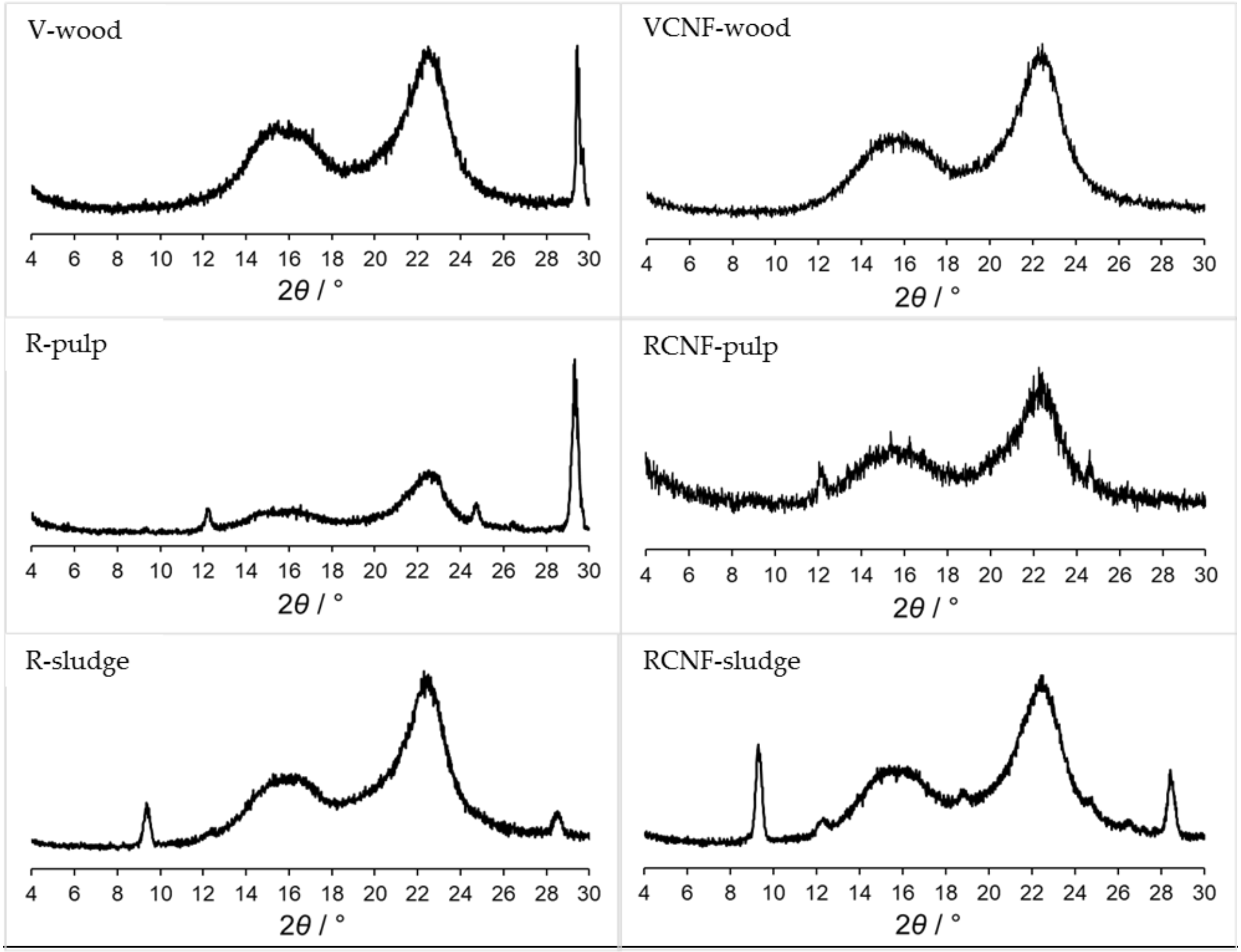


**Figure S8.** X-Ray diffractograms of cellulose sources and additives obtained by TOHO process.
